# The Use of Antenatal Dexamethasone in Late Preterm and Term Pregnancies to Improve Neonatal Morbidity and Mortality: A Systematic Review and Meta-Analysis

**DOI:** 10.7759/cureus.27865

**Published:** 2022-08-10

**Authors:** Alexandros Samouilidis, Eleftherios T Beltsios, Georgios Mavrovounis, Antonis Adamou, Ioannis Belios, Alexandros Hadjivasilis, Ioannis Pantazopoulos, Aris P Agouridis

**Affiliations:** 1 Internal Medicine, European University Cyprus, Nicosia, CYP; 2 Emergency Medicine, University of Thessaly, Larissa, GRC; 3 Radiology, University of Thessaly, Larissa, GRC; 4 Epidemiology and Public Health, Cyprus University of Technology, Limassol, CYP

**Keywords:** dexamethasone, antenatal dexamethasone, late preterm pregnancies, preterm birth, term pregnancies, cesarean section, neonatal respiratory distress, neonatal intensive care unit, transient tachypnea of newborn, meta-analysis

## Abstract

There are no acceptable worldwide recommendations regarding the use of dexamethasone in late-preterm newborns delivered either vaginally or via cesarean section and term gestation that are performed via cesarean section. The present study aims to compare the effectiveness of antenatal intramuscular dexamethasone versus placebo/no-treatment in reducing neonatal respiratory complications in high-risk for imminent preterm birth in late preterm pregnancies and term pregnancies undergoing elective cesarean section. The PubMed, Scopus, and Cochrane Library databases were searched to assess the effectiveness of dexamethasone during late preterm and term gestation. The last literature search was performed on March 20th, 2022. Randomized controlled trials compared antenatal dexamethasone administration with placebo or no treatment. The outcomes of interest were: the incidence of Respiratory Distress Syndrome; Transient Tachypnea of the Newborn, Neonatal Intensive Care Unit admissions; and the need for ventilatory support or mechanical ventilation.

A standardized data form and three independent investigators performed the data extraction. Ten RCTs fulfilled our inclusion criteria. No statistically significant difference was found regarding all of the outcomes in the 34th-36th gestational week group. In the >37th gestational week group, a statistically significant difference was found regarding the incidence of RDS [RR (95% CI); p-value: 0.56 (0.36, 0.87); 0.01], TTN [RR (95% CI); p-value: 0.54 (0.42, 0.71); <0.00001], need for ventilatory support [RR (95% CI); p-value: 0.71 (0.52, 0.96); 0.03] and need for mechanical ventilation [RR (95% CI); p-value: 0.56 (0.33, 0.95); 0.03]. To conclude, the antenatal administration of dexamethasone can be considered to prevent neonatal complications and reduce perinatal morbidity in term pregnancies.

## Introduction and background

Preterm birth (PTB) is the first cause of neonatal mortality and the second cause of death below the age of five years [1]. Although most preterm neonates survive, they remain at increased risk of neurodevelopmental impairments, respiratory complications, gastrointestinal complications, and neonatal infections [2]. Amongst respiratory complications, respiratory distress syndrome (RDS), a consequence of immature lung development, is the primary cause of early neonatal mortality and long-term morbidity in survivors [1]. Another commonly reported respiratory complication of preterm labor is transient tachypnea of the newborn (TTN) [2]. RDS and TTN may lead to an increased Neonatal Intensive Care Unit (NICU) admission rate [3].

The maternal use of steroids before preterm labor decreases complications related to the immaturity of the lungs and the absence of pulmonary surfactant [4]. Many Randomized Controlled Trials (RCTs) have been conducted, indicating the effectiveness of the antenatal use of corticosteroids in reducing neonatal complications in preterm labor [4]. Most guidelines recommend administering corticosteroids (dexamethasone or betamethasone) antenatally, especially in high-risk pregnancies for imminent PTB during the 24th to 34th week of gestation [5].

Cesarean section is a risk factor for developing neonatal respiratory distress [6,7]. It is suggested that elective or emergent cesarean section can increase the risk of newborns developing RDS, irrespective of the week of gestation. In most cases, the earlier the delivery week, the greater the risk for RDS [8]. However, recommendations for using corticosteroids to prevent neonatal respiratory complications in term gestations performed with cesarean section are lacking. Our study aimed to evaluate the effectiveness of antenatal intramuscular dexamethasone in decreasing the rate of neonatal RDS, TTN, and NICU admissions in late preterm pregnancies at high risk for imminent preterm birth as well as in term pregnancies undergoing elective cesarean section.

## Review

Materials and Methods

Protocol

The protocol for this systematic review and meta-analysis was registered on the International Prospective Register of Systematic Reviews (PROSPERO) with the id: CRD42021244108, and it is available in full https://www.crd.york.ac.uk/prospero/display_record.php?ID=CRD42021244108.

Search Strategy

Two authors (A.S. and A.A.) performed an electronic search of PubMed (MEDLINE) and Cochrane Library. The last literature search was performed on March 20th, 2022. The search algorithm included the following terms combined with the Boolean operators "AND" and "OR", as appropriate: "corticosteroids", "antenatal corticosteroids", "dexamethasone", "antenatal dexamethasone", "random", "controlled trial", "clinical trial", "randomized controlled trial", "placebo", "double-blind", "term", "late preterm", "Cesarean". No MeSH strategy was used. The exact search algorithms are presented in Appendix A. To identify additional studies that fulfilled our inclusion criteria, we manually searched the reference lists of the retrieved articles.

Inclusion and Exclusion Criteria

We included studies that: examined the administration of dexamethasone vs. placebo or no treatment in RCTs of pregnant women with singleton gestations that were either in the late preterm period (34th-36th gestational weeks) at high risk for preterm delivery or at the term period of pregnancy (>37th gestational weeks) and were scheduled for an elective cesarean section at this period.

We excluded studies that: involved multiple gestations, congenital malformed fetuses, intrauterine growth restriction fetuses (IUGR), women at less than 34 weeks of gestation, women that received prophylactic dexamethasone prior to the pregnancy, women with fever, chorioamnionitis, preeclampsia, known fetal anomaly, dexamethasone allergy, placenta previa, placenta abruption. In addition, we excluded clinical trials of women receiving antenatal betamethasone or multiple courses of antenatal corticosteroids. Furthermore, we excluded studies that did not implement an RCT design or explored non-relevant outcomes. Abstracts, reviews, and case report studies were also excluded.

Outcomes

The primary outcomes of the present study were the incidence of neonatal respiratory comorbidities (RDS and TTN). The secondary outcomes of this study were the incidence of NICU admission, the need for ventilatory support, and the need for mechanical ventilation.

Data Extraction

Three independent investigators (A.S., I.B., and A.A.) performed the data extraction using a standardized data form. We extracted the following data: First Author's Name, Year of Publication, Type of Study, Gestational week, study arm (dexamethasone, placebo/no treatment), dosage, and route of administration, when available. We also extracted the mean maternal age, the number of participants in each group, primary and secondary outcomes, and data regarding the cesarean sections performed in each study. Any discrepancy between the reviewers was resolved by a fourth investigator (ET.B.)

Quality Scoring and Publication Bias

The quality and methodological evaluation of the eligible studies were performed with the RoB 2.0 tool [9]. Two authors (A.A. and I.B.) independently assessed the selected articles for methodological quality. In case of disagreements between the two authors, a third author (ET.B.) offered his assessment to resolve the disagreement. In order to determine the possible presence of pub­lication bias, the funnel plot of the primary endpoint was visually inspected, and Egger's test was also performed for all outcomes.

Statistical Analysis

The comparison complication rates between the dexamethasone group and the control group were done by calculating the 95% confidence interval (95% CI) and the pooled risk ratios (RR) using an inverse variance method. The significance was set at P<0.05. The use of the Z test determined the statistical significance of the RR. To estimate the statistical heterogeneity of the studies, the I2 indices were calculated. The random effects model was applied when I2>50% [10]. Otherwise, the fixed effects model was used. Publication bias was assessed using funnel plots and Egger's test [11,12]; P values less than 0.05 indicate significant publication bias. All statistical analyses were performed in Review Manager (RevMan) [Computer program]. Version 5.3. Copenhagen: The Nordic Cochrane Centre, The Cochrane Collaboration, 2014 [13]. To investigate if differences in the dosage and the regimens affect the results, we performed a subgroup analysis when a sufficient number of studies were available in each subgroup for the outcome. Subgroup analyses were considered when at least three studies reported data for patients treated with either 8mg*3, 12mg*2, or 6mg*4 of dexamethasone. Sensitivity analyses were performed to assess the robustness of our results [14]. To investigate whether the inclusion of women beyond the 40th gestational week affects the significance of our results, we performed sensitivity analyses excluding the studies that included such patients.

Results

Selection and Characteristics of the Included Studies

**Figure 1:**
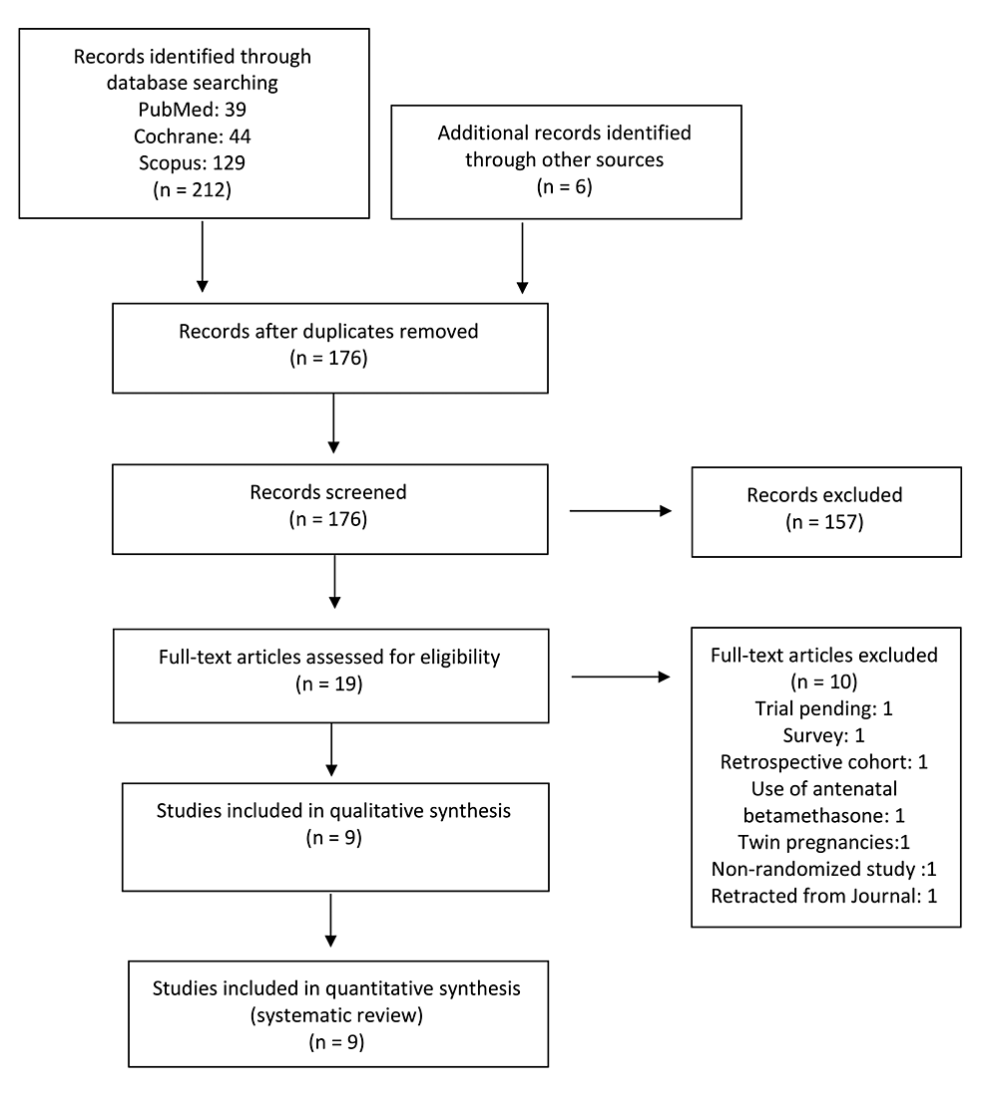
Study selection flow chart Flow diagram for study selection according to the PRISMA guidelines

The study was selected according to the Preferred Reporting Items for Systematic Reviews and Meta-Analyses (PRISMA) guidelines [15]. Our electronic database search resulted in 212 articles, and six additional records were identified through other sources (PubMed relevant articles, Google Scholar). After the duplicates were removed, 176 records were screened for inclusion based on their titles and abstracts. The full texts of the remaining 19 articles were reviewed, and nine articles that fulfilled our predetermined criteria were included in our systematic review and meta-analysis [16-24]. The flow chart for study selection according to PRISMA guidelines is shown in Figure 1.

Out of the nine studies, three included women at 34th-36th gestational weeks, while six included women beyond the 37th gestational week. The characteristics of the studies and the basic demographic information are presented in Table 1.

**Table 1:** Studies Characteristics The table presents the main characteristics of all the included studies. YOP: year of publication, RCT: randomized controlled trial, N/A: not applicable, T/C: treatment/control, IM: intramuscular, mg: milligrams, h: hours. * T: <19: 1.1%, 20-30: 44.5%, 31-40: 52.3%, >40: 2.1%; C: <19: 2.2%, 20-30: 49.1%, 31-40: 41.5%, >40: 2.1%

Author - YOP	Country	Study Design	n	Mean maternal age (T/C)	Treatment group	Control group	Lost to Follow-up (T/C)	Corticosteroid used	Route	Dosage
Late preterm pregnancies										
Ontela - 2018 [20]	India	RCT	310	24.3 / 24.1	154	155	1 / 0	Dexamethasone or nothing	IM	4 doses, 6mg, 12h
Attawattanakul - 2015 [19]	Thailand	RCT	194	24.7 / 26	96	98	5 / 0	Dexamethasone or no dexamethasone	IM	4 doses, 6mg, 12h or until delivery
Nabhan - 2014 [24]	Egypt	RCT	130	26.6 / 26.4	63	60	2 / 5	Dexamethasone or no dexamethasone	IM	4 doses, 12mg, 12h
Elective cesarean section in term pregnancies										
Elbohoty - 2020 [22]	Egypt	RCT	400	31.45 / 31.45	200	200	19 / 18	Dexamethasone or placebo (IM saline)	IM	4 doses, 6mg, 12h, at least 24h before c-section
Afzal - 2019 [16]	Pakistan	RCT	120	30 / 29.8	60	60	n/a	Dexamethasone or no dexamethasone	IM	2 doses, 12mg, 12h apart, 48h prior to cesarean section
Sadiq - 2019 [23]	Pakistan	RCT	320	30.5 / 29.4	158	162	6 / 10	Dexamethasone or no dexamethasone	IM	2 doses, 12mg, 12h
Ismail - 2017 [25]	Sudan	RCT	560	N/A *	281	279	n/a	Dexamethasone or no dexamethasone	IM	2 doses, 12mg, 12h or 48h before c-section
Nooh - 2017 [21]	Egypt	RCT	1400	26.2 / 25.9	700	700	64 / 64	Dexamethasone or no dexamethasone	IM	3 doses, 8mg, 8h, c-section after 24h
Ahmed - 2015 [17]	Egypt	RCT	452	27.5 / 28.2	228	224	n/a	Dexamethasone or no dexamethasone	IM	2 doses, 12mg, 24h

Outcome: 34th - 36th gestational weeks

Risk of Respiratory Distress Syndrome (RDS)

**Figure 2:**
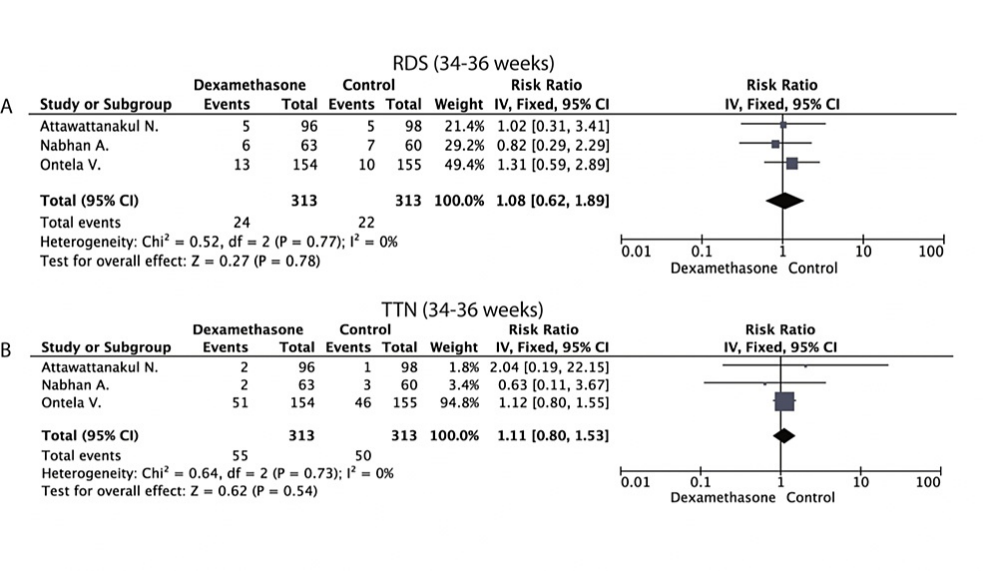
Forest plot Forest plots presenting the results regarding the development of (A) Respiratory Distress Syndrome and (B) Transient Tachypnea of neonates in the 34-36 gestational weeks group. Ontela - 2018 [19], Attawattanakul - 2015 [18], Nabhan - 2014 [23].

The heterogeneity of the included studies was found to be low, as indicated by the I2 index (I2=0%). Therefore, the fixed model was used. Our analysis revealed no statistically significant difference in the occurrence of RDS in the dexamethasone group compared to the control group [RR (95% CI): 1.08 (0.62, 1.89); P= 0.78]. The results of this analysis are presented in Figure 2A.

Transient Tachypnea of the Newborn (TTN)

Due to the low heterogeneity indicated by the I2 index (I2=0%), the fixed model was used. Our analysis showed no statistically significant difference in the occurrence of TTN in the dexamethasone group compared to the control group [RR (95% CI): 1.11 (0.80, 1.53); P=0.54]. The results of this analysis are presented in Figure 2B.

Admission to Neonatal Intensive care Unit (NICU)

Due to the low heterogeneity indicated by the I2 index (I2=14%), the fixed model was used. Our analysis showed no statistically significant difference in the rates of admission to NICU in the dexamethasone group compared to the control group [RR (95% CI): 1.05 (0.62, 1.78); P=0.85]. 

Need for Ventilatory Support

Due to the low heterogeneity indicated by the I2 index (I2=37%), the fixed model was used. Our analysis showed no statistically significant difference in the need for ventilatory support in the dexamethasone group compared to the control group [RR (95% CI): 0.75 (0.43, 1.31); P=0.31].

Need for Mechanical Ventilation

Due to the low heterogeneity indicated by the I2 index (I2=0%), the fixed model was used. Our analysis showed no statistically significant difference in the need for mechanical ventilation in the dexamethasone group compared to the control group [RR (95% CI): 0.63 (0.28, 1.43); P=0.27]. Table 1 summarizes the results of all analyses.

Outcome: >37th gestational weeks

Risk of Respiratory Distress Syndrome (RDS)

**Figure 3:**
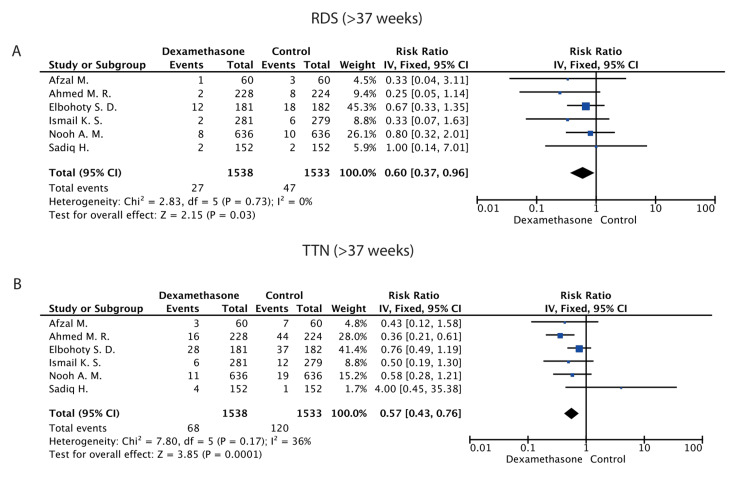
Forest plots Forest plots presenting the results regarding the development of (A) Respiratory Distress Syndrome and (B) Transient Tachypnea of neonates in the >37 gestational weeks group. Elbohoty - 2020 [21] , Afzal - 2019 [16] , Sadiq - 2019 [22] , Ismail - 2017 [24] , Nooh - 2017 [20] , Ahmed - 2015 [17]

Due to the low heterogeneity indicated by the I2 index (I2=0%), the fixed model was used. Our analysis revealed a statistically significant decrease in the occurrence of RDS in the dexamethasone group compared to the control group [RR (95% CI): 0.60 (0.37, 0.96); P= 0.030]. The results of this analysis are presented in Figure 3A.

Transient Tachypnea of the Newborn (TTN)

Due to the low heterogeneity indicated by the I2 index (I2=36%), The fixed model was used. Our analysis revealed a statistically significant reduction in the occurrence of TTN in the dexamethasone group compared to the control group [RR (95% CI): 0.57 (0.43, 0.76); P=0.0001]. The results of this analysis are presented in Figure 3B.

Admission to Neonatal Intensive care Unit (NICU)

Due to the low heterogeneity indicated by the I2 index (I2=71%), the random model was used. Our analysis showed no statistically significant difference in the rates of admission to NICU in the dexamethasone group compared to the control group [RR (95% CI): 0.56 (0.24, 1.32); P=0.18].

Need for Ventilatory Support

Due to the low heterogeneity indicated by the I2 index (I2=19%), the fixed model was used. Our analysis showed no statistically significant difference in the need for ventilatory support in the dexamethasone group compared to the control group [RR (95% CI): 0.74 (0.54, 1.02); P=0.06]. 

Need for Mechanical Ventilation

Due to the low heterogeneity indicated by the I2 index (I2=0%), the fixed model was used. Our analysis showed no statistically significant difference in the need for mechanical ventilation in the dexamethasone group compared to the control group [RR (95% CI): 0.60 (0.33, 1.12); P=0.11]. Table 2 summarizes the results of all analyses.

**Table 2:** Summary of primary and subgroup analyses Table summarizing the results of all primary and subgroup analyses. Abbreviations. RR: Risk Ratio; CI: Confidence Interval; RDS: Respiratory Distress Syndrome; TTN: Transient Tachypnea of neonates; NICU: Neonatal Intensive Care Unit

			Dexamethasone group		Control group			
Outcome	Gestational age (weeks)	Refs	Events (n)	Total (n)	Events (n)	Total (n)	RR (95% CI)	p value
RDS	34-36	18, 19, 23	24	313	22	313	1.08 (0.62, 1.89)	0.78
RDS	>37	16, 17, 20-22, 24	27	1538	47	1533	0.60 (0.37, 0.96)	0.03
RDS dosage subgroup analysis (12mg*2)	>37	16, 17, 20-22, 24	7	721	19	715	0.38 (0.16, 0.91)	0.03
TTN	34-36	18, 19, 23	55	313	50	313	1.11 (0.80, 1.53)	0.54
TTN	>37	16, 17, 20-22, 24	68	1538	120	1533	0.57 (0.43, 0.76)	0.0001
TTN dosage subgroup analysis (12mg*2)	>37	16, 17, 20-22, 24	29	721	64	715	0.43 (0.28, 0.66)	0.0001
NICU admission	34-36	18, 19, 23	26	313	25	313	1.05 (0.62, 1.78)	0.85
NICU admission	>37	16, 17, 20, 22	37	1076	57	1072	0.56 (0.24, 1.32)	0.18
NICU admission dosage subgroup analysis (12mg*2)	>37	16, 17, 20, 22	24	440	36	436	0.49 (0.12, 1.94)	0.31
Ventilatory support	34-36	18, 19, 23	21	313	28	313	0.75 (0.43, 1.31)	0.31
Ventilatory support	>37	16, 20-22	53	1029	71	1030	0.74 (0.54, 1.02)	0.06
Mechanical ventilation	34-36	18, 19, 23	9	313	15	313	0.63 (0.28, 1.43)	0.27
Mechanical ventilation	>37	16, 20-22	11	1029	26	1030	0.60 (0.33, 1.12)	0.11

Publication Bias and Study Quality Assessment

**Figure 4:**
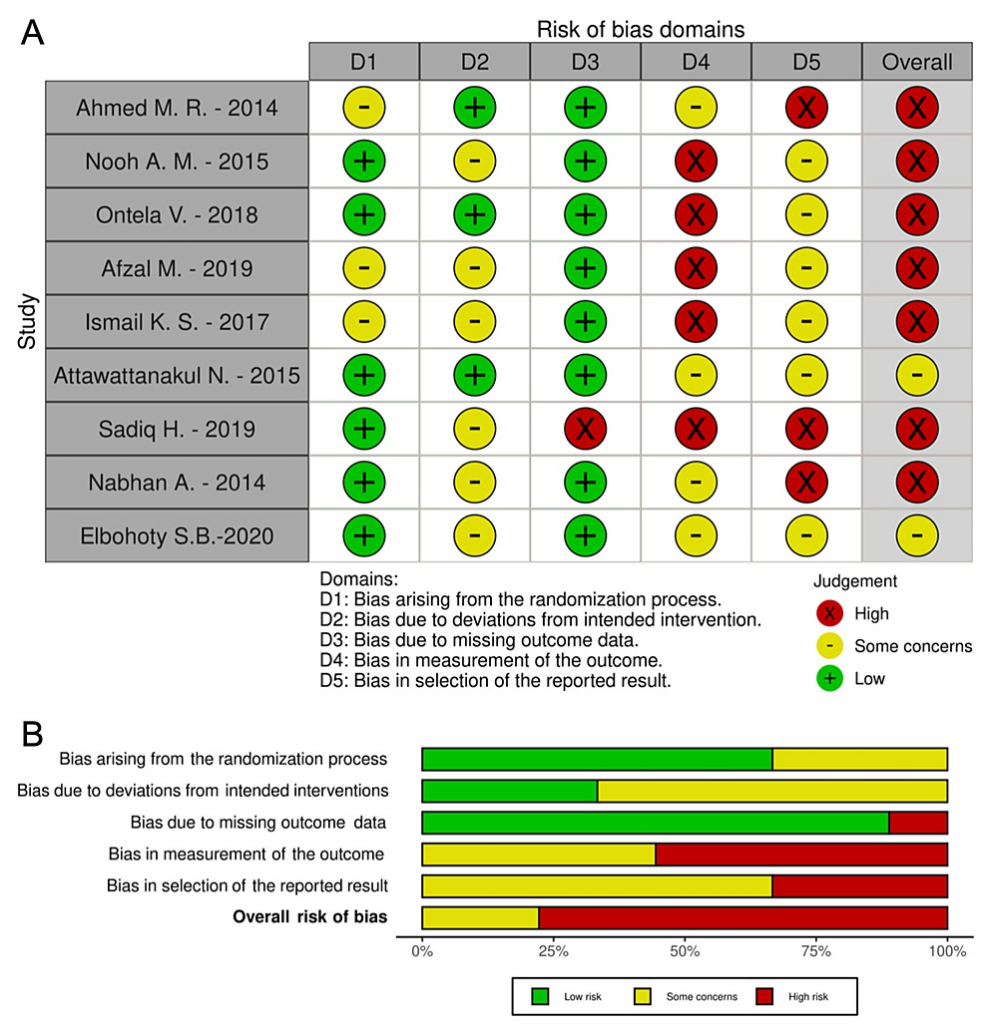
Bias assessment The figure for the visualization of the risk of bias assessment (A) for each individual study and (B) overall, using the RoB 2.0 tool. Ontela - 2018 [19], Attawattanakul - 2015 [18], Nabhan - 2014 [23], Elbohoty - 2020 [21], Afzal - 2019 [16], Sadiq - 2019 [22], Ismail - 2017 [24], Nooh - 2017 [20], Ahmed - 2015 [17].

The overall risk of publication bias was high according to the quality and methodological evaluation of the eligible studies performed with the RoB 2 tool. Seven out of the nine included studies were evaluated as "high risk," while 2 out of the 10 studies scored a neutral result indicating "some concerns". The primary sources of the high publication bias are related to the measurement of the outcome and the selection of the reported results (D4 and D5 domains of the RoB 2 tool, respectively). The results of the study quality assessment are summarized in Figure 4.

**Figure 5:**
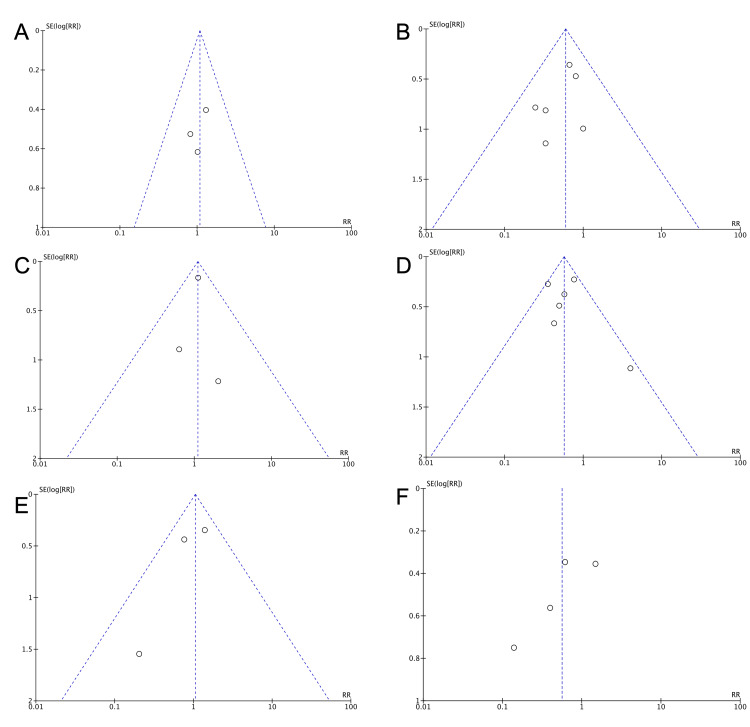
Funnel plots for the presence of publication bias Funnel plots for studying the presence of publication bias for the analyses: (A) RDS in the 34-36 gestational weeks group, (B) RDS in the >37 gestational weeks group, (C) TTN in the 34-36 gestational weeks group, (D) TTN in the >37 gestational weeks group, (E) NICU admission in the 34-36 gestational weeks group, (F) NICU admission in the >37 gestational weeks group. Abbreviations: RDS: Respiratory Distress Syndrome; TTN: Transient Tachypnea of neonates; NICU: Neonatal Intensive Care Unit.

**Figure 6:**
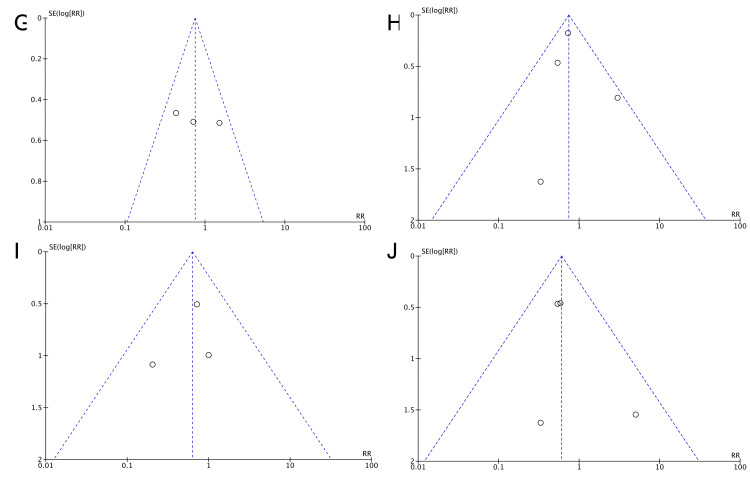
Funnel plots for the presence of publication bias Funnel plots for studying the presence of publication bias for the analyses: (G) Need for ventilatory support in the 34-36 gestational weeks group, (H) Need for ventilatory support in the >37 gestational weeks group, (I) Need for mechanical ventilation in the 34-36 gestational weeks group, (J) Need for mechanical ventilation in the >37 gestational weeks group.

Visual inspection of the funnel plots to identify possible publication bias (Figure 5, 6) did not reveal any significant asymmetry. This was confirmed by the Egger's and Begg's tests (Table 3).

**Table 3:** Egger and Begg Test. Abbreviations. RDS: Respiratory Distress Syndrome; TTN: Transient Tachypnea of neonates; NICU: Neonatal Intensive Care Unit

Outcome	Gestational age (weeks)	Egger (p-value)	Begg (p-value)
RDS	34-36	0.491	0.602
RDS	>37	0.277	0.851
TTN	34-36	0.951	0.602
TTN	>37	0.658	0.851
NICU admission	34-36	0.360	0.117
NICU admission	>37	0.099	0.174
Ventilatory support	34-36	0.329	0.117
Ventilatory support	>37	0.796	1.000
Mechanical ventilation	34-36	0.692	0.602
Mechanical ventilation	>37	0.496	0.497

Subgroup Analysis

When dosage subgroup analysis was performed in the >37th gestational weeks group regarding the risk of neonatal RDS, the use of 2 doses of 12mg of dexamethasone maintained a statistically significant favorable outcome when compared to the placebo/ no treatment group [RR (95% CI): 0.38 (0.16, 0.91); P=0.03].

When dosage subgroup analysis was performed in the >37th gestational weeks group regarding the risk of TTN, the 12mg * 2 dosages maintained a statistically significant favorable outcome when compared to the placebo/ no treatment group [RR (95% CI): 0.43 (0.28, 0.66); P=0.0001]. The results of the subgroup analysis are presented in Table 2.

Sensitivity Analysis

The sensitivity analysis proved the robustness of our results regarding the primary outcomes. Regarding the secondary outcomes, the sensitivity analysis revealed a non-statistically significant difference [RR (95%): 0.86 (0.21, 3.51); P=0.83] in need for ventilatory support between the two groups. Furthermore, a non-statistically significant difference [RR (95%): 0.56 (0.22, 1.43); P=0.22] was found regarding the need for mechanical ventilation between the two groups. Table 4 summarizes the results of the sensitivity analyses.

**Table 4:** Results of the sensitivity analyses Table summarizing the results of the sensitivity analyses. Abbreviations. RR: Risk Ratio; CI: Confidence Interval; RDS: Respiratory Distress Syndrome; TTN: Transient Tachypnea of neonates; NICU: Neonatal Intensive Care Unit

Outcome*	RR (95% CI)	p value
RDS	0.38 (0.19, 0.77)	0.008
TTN	0.42 (0.28, 0.61)	<0.00001
NICU admission	0.49 (0.19, 1.24)	0.13
Ventilatory support	0.86 (0.21, 3.51)	0.83
Mechanical ventilation	0.56 (0.22, 1.43)	0.22

Discussion

The present study confirmed a statistically significant lower risk of RDS (P=0.03) and TTN (P<0.01) in neonates born beyond the 37th gestational week and had received antenatal dexamethasone seven days prior to labor. Statistical analysis did not reveal any significant association between dexamethasone and the risk of NICU admission, the need for mechanical ventilation, or ventilatory support in the same group. Additionally, neonates born between the 34th and 36th gestational weeks revealed no statistically significant association with any studied outcomes. Subgroup analysis revealed an association of 12mg of dexamethasone twice daily with a lower risk of RDS and TTN in laborers exceeding the 37th gestational weeks.

A previous meta-analysis by Roberts et al. [4], which included 7774 women at risk of preterm birth and 8158 neonates (30 studies), supported using a single course of antenatal corticosteroids to accelerate fetal lung maturation. Another meta-analysis [25] that assessed the effect of prophylactic antenatal corticosteroid administration prior to term cesarean sections in 3956 women and 3893 neonates (4 trials) reported a reduction of RDS, TTN, and NICU admission for respiratory morbidity.

A great debate was found in studies of newborns born during the late preterm period who needed ventilatory support. A randomized controlled trial [19] reported that antenatal dexamethasone did not provide a statistically significant reduction in RDS and TTN. Likewise, Nabhan et al. [23] concluded that dexamethasone did not reduce admission to NICU, RDS, TTN, need for mechanical ventilation, development of respiratory or non-respiratory complications, and readmission for respiratory problems during the late preterm period in neonates born through a cesarean section. Besides, Attawattanakul et al. [18] investigated the effect of dexamethasone in late preterm pregnancies. They reported a statistically significant reduction in the rate of RDS owing to dexamethasone, while results for TTN, NICU admission, and the need for ventilatory support showed no statistical significance.

The glucocorticoid receptor is a critical player in the whole process of lung maturity. Glucocorticoids bind to the receptor inducing a series of morphological alterations, including a change in the mesenchymal tissue, which thins markedly [26]. Endogenous corticosteroid production in embryos is boosted following the marked decline of 11β-hydroxysteroid dehydrogenase-2 (11β-HSD-2) in fetal serum, which coincides with the marked increase of maternal serum glucocorticoids [27]. Like any other artificial corticosteroid, the administration of dexamethasone aims to enhance the production of the surfactant factor in preterm neonates, as the endogenous corticosteroids would do in physiological circumstances. Although dexamethasone was administered in varying schemes between the included studies, the World Health Organization (WHO) recommends 24mg of dexamethasone in divided doses and a single repeated dose if labor has not been managed by the course of 7 days [28].

In term cesarean sections, a study by Ahmed et al. [17] showed a statistically significant reduction of TTN and RDS in neonates after antenatal administration of dexamethasone in women undergoing elective term cesarean section and reported that the administration of dexamethasone at the 37th gestational week presented the maximum effect in reducing respiratory complications. Furthermore, a clinical trial that included 120 women [16] concluded that elective cesarean should be delayed up to 39 weeks. If an early-term elective section is required, prophylactic dexamethasone 48 hours prior to cesarean section reduces neonatal respiratory morbidity and can be safely used. Moreover, a study conducted in Sudan [24] reported that dexamethasone 48 hours before a scheduled cesarean section significantly decreased neonatal respiratory complications and NICU admissions. Notably, no infant from the treatment group born later than the 39th gestational week was admitted to NICU. Contrariwise, Nooh et al. [20] did not find any statistically significant reduction in neonatal RDS, TTN, NICU admission rates, and need for respiratory support following antenatal corticosteroids. Likewise, Sadiq et al. [22] reported that dexamethasone had no significant effect on decreasing the rate of respiratory complications, NICU admissions, and the need for respiratory support. A recent single-center study reported that more than half the studied neonates admitted to the NICU were preterm, with most of them being late-preterm neonates [29]. Additionally, they concluded that prematurity, RDS, and TTN were the main indications for NICU admission.

Limitations

The present study has several limitations that should be addressed. The exclusion of grey literature in our data acquisition strategy raises concerns about possible publication bias. Therefore, the results of our analysis should be interpreted cautiously since treatment effects might be overestimated in meta-analyses, including only published data [30]. Furthermore, our study included a small number of participants and studies with a high drop-out rate. Additionally, some studies did not report the number of infants who required respiratory support. There are also some limitations arising from the design of the included studies, resulting in a confined result regarding the quality assessment of the included studies. Lastly, our study is subject to language bias since only studies written in English were included.

## Conclusions

Our results indicate that the antenatal administration of corticosteroids can be considered to prevent neonatal RDS and TTN, thus reducing perinatal morbidity arising from cesarean sections in terminal pregnancies. No effect was observed in late preterm pregnancies; however, further research is needed to adequately assess the role of antenatal dexamethasone in preventing premature neonatal complications in the late preterm period.

## Appendices

Appendix A 

The exact search algorithms that were implemented during the database search

PubMed

(corticosteroids* OR antenatal corticosteroids OR dexamethasone OR antenatal dexamethasone) AND (“random*” OR “controlled trial” OR “clinical trial” OR “randomized controlled trial” OR “placebo” OR “double-blind”) AND (term OR late preterm) AND (Cesarean) in All Fields

Cochrane Library

corticosteroids* OR antenatal corticosteroids OR dexamethasone OR antenatal dexamethasone in Title Abstract Keyword AND “random*” OR “controlled trial” OR “clinical trial” OR “randomized controlled trial” OR “placebo” OR “double-blind” in Title Abstract Keyword AND term OR late preterm in Title Abstract Keyword AND Cesarean in Title Abstract Keyword.
